# Improving multiple model ensemble predictions of daily precipitation and temperature through machine learning techniques

**DOI:** 10.1038/s41598-022-08786-w

**Published:** 2022-03-18

**Authors:** Dinu Maria Jose, Amala Mary Vincent, Gowdagere Siddaramaiah Dwarakish

**Affiliations:** 1 Department of Water Resources and Ocean Engineering, National Institute of Technology Karnataka, Surathkal, Mangaluru, India; 2 Department of Mathematical and Computational Sciences, National Institute of Technology Karnataka, Surathkal, Mangaluru, India

**Keywords:** Climate change, Hydrology

## Abstract

Multi-Model Ensembles (MMEs) are used for improving the performance of GCM simulations. This study evaluates the performance of MMEs of precipitation, maximum temperature and minimum temperature over a tropical river basin in India developed by various techniques like arithmetic mean, Multiple Linear Regression (MLR), Support Vector Machine (SVM), Extra Tree Regressor (ETR), Random Forest (RF) and long short-term memory (LSTM). The 21 General Circulation Models (GCMs) from National Aeronautics Space Administration (NASA) Earth Exchange Global Daily Downscaled Projections (NEX-GDDP) dataset and 13 GCMs of Coupled Model Inter-comparison Project, Phase 6 (CMIP6) are used for this purpose. The results of the study reveal that the application of a LSTM model for ensembling performs significantly better than models in the case of precipitation with a coefficient of determination (R^2^) value of 0.9. In case of temperature, all the machine learning (ML) methods showed equally good performance, with RF and LSTM performing consistently well in all the cases of temperature with R^2^ value ranging from 0.82 to 0.93. Hence, based on this study RF and LSTM methods are recommended for creation of MMEs in the basin. In general, all ML approaches performed better than mean ensemble approach.

## Introduction

Accurate climate prediction is vital in planning and management of water resources for long-term sustainability of hydrological projects^1^. Global Circulation Models (GCMs) are considered to be the most dependable numerical models for understanding likely future climate^2,3^. GCMs simulate the past climate based on the observed concentrations of greenhouse gases (GHGs) and simulate the likely future climate based on the given likely future concentrations of GHGs^4^. However, uncertainties are involved in the past and future simulations made by GCMs even after significant improvements made in the recent versions of GCMs^5,6^. Knowledge of possible uncertainties and their respective solutions have also significantly progressed through the years^3,5,7,8^.

A watershed level climate analysis is necessary for planning suitable mitigation and adaptation techniques^9,10^. The GCMs are often incapable of giving fine scale simulations which are required for local scale studies. In order to overcome this limitation, several downscaling techniques are developed and improved^11,12^. However these downscaled or raw simulations of GCMs often have considerable bias, which are frequently corrected through appropriate bias correction techniques^13–16^. Another strategy used for reducing the uncertainties associated with GCMs is through appropriate selection of GCM/GCMs^3,17^. Different approaches are followed for the selection of the best GCM or an ensemble of GCMs.

Many of the earlier studies used the outputs of a single GCM. Recently, usage of ensembles of several GCMs have become a common practice^18^. The main aim of using multi-model ensembles (MMEs) / ensemble is to improve the reliability of future projections^19^. In general, ensembling is done in two ways: (1) Calculating the mean or median of a set of GCM outputs, and (2) Giving weights to different GCMs considered. In order to calculate the weights of the GCMs according to their performance in the past, multi-criteria decision making methods or other matrices are often used^3,20^. Multiple linear regressions (MLR) to complex machine learning (ML) algorithms are used to develop MMEs. ML algorithms are gaining popularity since they are found to be more effective compared to other techniques of ensembling (e.g. Acharya et al.^21^; Ahmed et al.^2^; Crawford et al.^22^; Sachindra et al.^23^; Wang et al.^24^). However, most of these studies have done monthly, annual or seasonal evaluation. Thus, reliable MMEs of daily climate variables are thus necessary.

All the above approaches basically consider climate data to be stationary and linear. Several ML models have been proposed for climate data downscaling and multi-model ensemble predictions as an alternative to these approaches that can address the non-linearity in time series data^2,23,25–32^. The most commonly used models are Support Vector Machine (SVM), Random Forest (RF) and the artificial neural networks (ANNs), which can model complex, mostly nonlinear relationships in climate data. Although these approaches can address non-linearity in the data, they have the drawback of assuming that all inputs and outputs are independent of each other, even when dealing with sequential data^33^. Since climate data has dependency between successive values, it is imperative to consider this dependency. Long Short-Term Memory (LSTM) deep learning models are designed specially to learn long-term dependencies present in sequential data^34^. Compared to shallow ANN architecture, deep learning models are more capable of representing highly varying nonlinear functions like complex temporal patterns via high-level temporal abstractions^35,36^.

The present study aims on comparison and improvement of MMEs using various ensembling techniques. In this objective, special attention is paid to improve the MMEs of daily climate variables like precipitation (P), minimum temperature (Tmin) and maximum temperature (Tmax). Furthermore, special emphasis has been given in testing the ability of each ensembling technique in simulating monsoon rainfall. The methodology proposed in the present study is demonstrated on Netravati basin, a tropical river basin on the southwest coast of India. The present paper is organized as follows: “Study area” and “Data products” sections, introduces the study area and datasets considered. “Methodology” section describes the related methodology followed for creating ensembles of GCMs using simple statistical techniques (mean), regression models (i.e., SVM and MLR), an ensemble learning models (i.e., extra tree regressor (ETR) and RF), and deep learning time series model (i.e., multivariate LSTM). “Results and discussion” section presents the results, while “Summary and conclusions” section concludes and discusses the scope for future work.

## Study area

**Figure 1:**
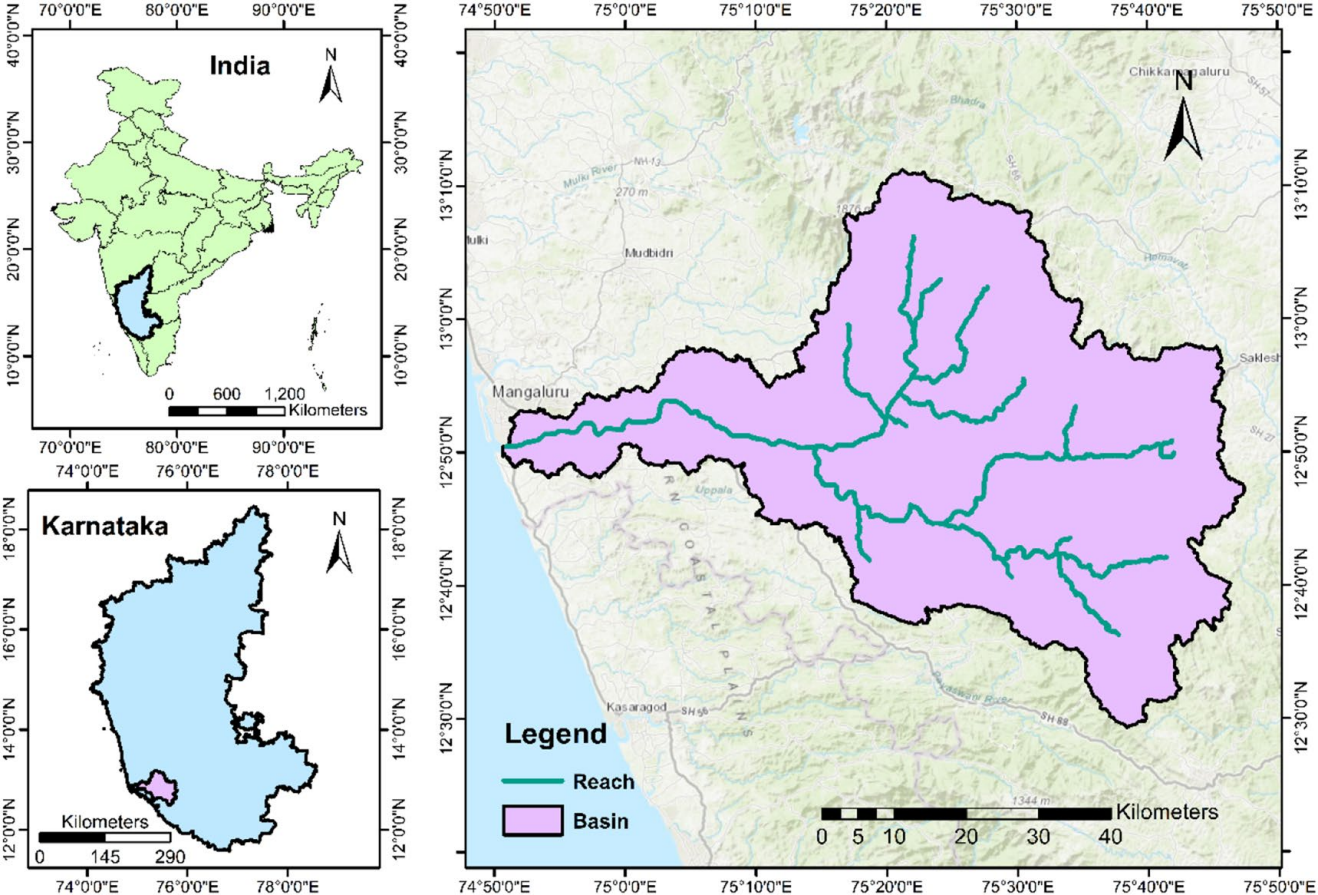
Location of the selected study area—Netravati basin (Generated using ArcMap 10.3).

The Western Ghats of India is one among the global biodiversity hotspots. It is biologically rich and biogeographically unique with diverse species of plants, mammals, birds and amphibians^37^. Netravati, a west-flowing river which drains into the Arabian Sea is located in the central zone of Western Ghats of India. This river is situated between 12°30′N and 13°10′N latitudes and 74°50′E and 75°50′E longitudes covering an area of about 3415 km^2^ (Fig. 1). The Netravati river basin experiences a humid tropical climate with an average annual rainfall of around 4000 mm. The rainfall over the basin is distributed into three seasons namely, Pre-Monsoon (March–May), Southwest Monsoon (June–September), and Northeast Monsoon (October–December). The Southwest monsoon is the major contributor to annual rainfall. The average daily temperature is the highest during March to May and lowest during December and January. The average minimum and maximum temperatures of the basin are about 19 and 29 °C, respectively. The elevation in the basin varies from 0 to 1884 m with respect to the MSL. Geologically, the basin is of Precambrian formations. The upper part of the basin mainly consists of sandy clay loam soil, while the lower parts consist of clay loam soil^38^. Mountainous dense forests cover the upstream parts of the basin while agricultural and urban lands dominate the lower parts. Netravati river is a major source of water for agriculture, industries and civic life in cities like Mangaluru, Bantwal, Puttur, Dharmasthala, Ujire etc. in the basin^39^. The basin is socially, economically and culturally important.

## Data products

### Reference precipitation and temperature dataset

High resolution gridded precipitation data from the year 1901 at a daily timescale with a spatial resolution of 0.25° longitude × 0.25° latitude has been made available by India Meteorological Department (IMD). This dataset is created by converting 6995 station-based observations into grid values using Shepard’s interpolation technique^40^. This dataset can represent the spatial rainfall distribution like heavy rainfall areas in the orographic regions of the west coast and low rainfall in the leeward side of the Western Ghats^40^. IMD also provides gridded daily maximum and minimum temperature from the year 1951 at a spatial of 1° longitude × 1° latitude. This dataset is developed based on the data from 395 quality-controlled stations using a modified version of the Shepard’s angular distance weighting algorithm for interpolation^41^. These datasets can be accessed through IMD Pune’s website (http://www.imdpune.gov.in/Clim_Pred_LRF_New/Grided_Data_Download.html). These datasets are extensively used for climate-related research and applications over India^42–45^. Hence, the daily rainfall and temperature dataset from the India Meteorological Department (IMD) was used in this study as the reference/observation dataset.

### GCM precipitation and temperature dataset

The statistically downscaled and bias-corrected Coupled Model Inter-comparison Project, Phase 5 (CMIP5) dataset provided by the National Aeronautics Space Administration (NASA) Earth Exchange Global Daily Downscaled Projections (NEX-GDDP) was used in this study. This dataset includes historical (1950–2005) and future (2006–2100) climate projections (Representative Concentration Pathways (RCP) scenarios 4.5 and 8.5) of precipitation and temperature at high spatial (25 km^2^ grid-scale) and temporal (daily) resolutions from 21 GCMs. The dataset is generated using Bias-Correction Spatial Disaggregation (BCSD) method^46^ using the Global Meteorological Forcing Dataset (GMFD) provided by the Terrestrial Hydrology Research Group at Princeton University^47^. This data can be accessed from NASA Centre for Climate Simulation (NCCS) portal (https://portal.nccs.nasa.gov/datashare/NEXGDDP/). The list of 21 GCMs with the country of their origin is given in Table 1. This dataset has been utilized for many studies around the world^42,47–49^. It is considered as the highest resolution and most accurate climate data based on CMIP5 scenarios in India^50^. The high resolution of NEX-GDDP not only provides information at finer scales but also incorporates local topography effects which influence the local extremes of rainfall events. A study by Jain et al., (2019) evaluated and compared the performance of NEX-GDDP dataset with CMIP5 and CORDEX datasets in India. They found that the NEX-GDDP data could realistically capture the precipitation and temperature variability in India and recommended it for future climate impact studies.

**Table 1:** Twenty-one CMIP5 models included in NEX-GDDP dataset.

Model name	Country	Latitude resolution (degree)	Longitude resolution (degree)	Description	Institution/Agency
ACCESS1-0	Australia	1.25	1.875	Australian Community Climate and Earth System Simulator, version 1.0	Commonwealth Scientific and Industrial Research Organisation (CSIRO) and Bureau of Meteorology (BoM)
BNU-ESM	China	2.8	2.8	Beijing Normal University Earth System Model	College of Global Change and Earth System Science and Beijing Normal University
CCSM4	United States	0.94	1.25	Community Climate System Model (CCSM), version 4	University Corporation for Atmospheric Research
CESM1-BGC	United States	0.94	1.25	Community Earth System Model, version 1–Biogeochemistry	National Science Foundation, Department of Energy, National Centre for Atmospheric Research
CNRM-CM5	France	1.4	1.4	Centre National de Recherches Météorologiques Coupled Global Climate Model, version 5	Centre National de Recherches Meteorologiques / Centre Europeen de Recherche et Formation Avancees en Calcul Scientifique
CSIRO-Mk3-6–0	Australia	1.8	1.8	Commonwealth Scientific and Industrial Research Organisation Mark 3.6.0	Queensland Climate Change Centre of Excellence and the Commonwealth Scientific and Industrial Research Organisation (CSIRO)
CanESM2	Canada	2.8	2.8	Second generation Canadian Earth System Model	Canadian Center for Climate Modelling and Analysis
GFDL-CM3	United States	2.0	2.5	Geophysical Fluid Dynamics Laboratory- Climate Model version 3	Geophysical Fluid Dynamics Laboratory
GFDL-ESM2G	United States	2.0	2.5	Geophysical Fluid Dynamics Laboratory Earth System Model with (GOLD) component	Geophysical Fluid Dynamics Laboratory
GFDL-ESM2M	United States	2.0	2.5	Geophysical Fluid Dynamics Laboratory Earth System Model with Modular Ocean Model (MOM), version 4 component	Geophysical Fluid Dynamics Laboratory
IPSL-CM5A-LR	France	1.8	3.75	L’Institut Pierre-Simon Laplace Coupled Model, version 5A, low resolution	Institut Pierre-Simon Laplace
IPSL-CM5A-MR	France	1.25	2.5	L’Institut Pierre-Simon Laplace Coupled Model, version 5A, mid resolution	Institut Pierre-Simon Laplace
MIROC-ESM	Japan	2.8	2.8	Model for Interdisciplinary Research on Climate, Earth System Model	Japan Agency for Marine-Earth Science and Technology, Atmosphere and Ocean Research Institute (The University of Tokyo), and National Institute for Environmental Studies
MIROC-ESM-CHEM	Japan	2.8	2.8	Model for Interdisciplinary Research on Climate, Earth System Model, Chemistry Coupled	Japan Agency for Marine-Earth Science and Technology, Atmosphere and Ocean Research Institute (The University of Tokyo), and National Institute for Environmental Studies
MIROC5	Japan	1.4	1.4	Model for Interdisciplinary Research on Climate, version 5	Japan Agency for Marine-Earth Science and Technology, Atmosphere and Ocean Research Institute (The University of Tokyo), and National Institute for Environmental Studies
MPI-ESM-LR	Germany	1.9	1.9	Max Planck Institute Earth System Model, low resolution	Max Planck Institute for Meteorology
MPI-ESM-MR	Germany	1.9	1.9	Max Planck Institute Earth System Model, medium resolution	Max Planck Institute for Meteorology
MRI-CGCM3	Japan	1.1	1.1	Meteorological Research Institute Coupled Atmosphere–Ocean General Circulation Model, version 3	Meteorological Research Institute
NorESM1-M	Norway	1.9	2.5	Norwegian Earth System Model 1-M	Norwegian Climate Centre
BCC − CSM1.1	China	2.8	2.8	Beijing Climate Center, Climate System Model, version 1.1	Beijing Climate Centre
INM-CM4	Russia	1.5	2.0	Institute of Numerical Mathematics Coupled Model, version 4	Russian Institute of Numerical Mathematics

Further, bias-corrected daily projections of precipitation, maximum temperature, and minimum temperature for South Asia developed by Mishra et al.^51^ using outputs from 13 CMIP6 GCMs was also used in this study. This dataset is bias-corrected using Empirical Quantile Mapping (EQM) for the historic (1951–2014) and projected (2015–2100) period. The dataset contains bias corrected projections for the four scenarios (SSP126, SSP245, SSP370, SSP585). This bias-corrected dataset is technically validated against the observations for both mean and extremes of precipitation, maximum and minimum temperatures^51^. Spatial resolution of this bias corrected dataset is 0.25°. The list of these 13 GCMs with the country of their origin is given in Table 2. Here after these GCMs are collectively referred to as CMIP6 dataset.

**Table 2:** Thirteen CMIP6 models considered in the study.

Model name	Country	Latitude resolution (degree)	Longitude resolution (degree)	Description	Institution/Agency
ACCESS-CM2	Australia	1.25	1.875	Australian Community Climate and Earth System Simulator Climate Model Version 2	Commonwealth Scientific and Industrial Research Organisation (CSIRO), Australian Research Council Centre of Excellence for Climate System Science (ARCCSS), and Bureau of Meteorology
ACCESS-ESM1-5	Australia	1.25	1.875	Australian Community Climate and Earth System Simulator Earth System Model Version 1.5	Commonwealth Scientific and Industrial Research Organisation (CSIRO)
BCC-CSM2-MR	China	1.1215	1.125		Beijing Climate Centre
CanESM5	Canada	2.7906	2.8125	Fifth generation Canadian Earth System Model	Canadian Centre for Climate Modelling and Analysis
EC-Earth3	Europe	0.7018	0.703125	EC-Earth Earth System Model Version 3	EC-Earth Consortium
EC-Earth3-Veg	Europe	0.7018	0.703125	EC-Earth Earth System Model Version 3 with Dynamic vegetation component	EC-Earth Consortium
INM-CM4-8	Russia	1.5	2	Institute of Numerical Mathematics Coupled Model, version 4.8	Russian Institute of Numerical Mathematics, Russian Academy of Science
INM-CM5-0	Russia	1.5	2	Institute of Numerical Mathematics Coupled Model, version 5	Russian Institute of Numerical Mathematics, Russian Academy of Science
MPI-ESM1-2-HR	Germany	0.9351	0.9375	Max Planck Institute for Meteorology Earth System Model version 1.2 higher resolution	Max Planck Institute for Meteorology
MPI-ESM1-2-LR	Germany	1.8653	1.875	Max Planck Institute for Meteorology Earth System Model version 1.2 low resolution	Max Planck Institute for Meteorology
MRI-ESM2-0	Japan	1.1215	1.125	Meteorological Research Institute Earth System Model Version 2.0	Meteorological Research Institute
NorESM2-LM	Norway	1.8947	2.5	Norwegian Earth System Model version 2 with low resolution atmosphere/land and medium resolution ocean/sea ice	Norwegian Climate Consortium (NCC)
NorESM2-MM	Norway	0.9424	1.25	Norwegian Earth System Model version 2 with medium resolution of both atmosphere/land and ocean/sea ice	Norwegian Climate Consortium (NCC)

## Methodology

There are many methods available for ensembling, like Bayesian approaches and machine learning approaches^52,53^. Six techniques were used for creating MMEs of P, Tmax and Tmin simulated by 21 NEX-GDDP and 13 CMIP6 GCMs in Netravati basin. These methods were mean, Multiple Linear Regression (MLR), Support Vector Machine (SVM), Extra Tree Regressor (ETR), Random Forest (RF) and long short-term memory (LSTM). These methods cover the major types of existing machine learning ensembling methods. These ensembling techniques can be classified as simple statistical techniques (mean), regression models (i.e., SVM and MLR), ensemble learning models (i.e., ETR and RF), and deep learning time series model (i.e., multivariate LSTM). All these methods try to improve the GCM simulations with respect to the observation dataset in the historical time period. All the BC methods except LSTM were implemented P, Tmin and Tmax using the scikit-learn library in Python^54^. The LSTM was implemented using Keras, which is one of the most popular deep learning libraries in Python^55^. All the calculations have been carried out independently for each grid cell and the results for one representative grid in the basin is shown to simplify the presentation. More about data pre-processing and a brief description of each ensembling method are provided in the following sections.

### Data preparation

Each ensembling method was carried out at each grid point considering P, Tmax and Tmin separately. Bilinear interpolation was done in order to bring the GCM values to the corresponding observation grids in the basin. Ensemble mean was calculated by finding the mean of P, Tmax and Tmin simulated by all GCMs at each grid respectively. The data was split into training and testing datasets for validation and comparison of each method of ensembling. The input to each ML model was preprocessed using Principal component analysis (PCA). More about PCA is described below.

### Principal component analysis (PCA)

Before applying any ML algorithm, it is vital to acquire only the relevant features in the training dataset. This way of reducing the feature space is termed as dimensionality reduction or feature selection^56^. In this study the features are the various GCMs in ensemble. Ahmed et al.^2^ has mentioned that choice of the number of the GCMs used in MME is a key decision in ensembling. In the present study PCA was used for this purpose. It is a part of the exploratory data analysis in ML technique for predictive models^57^. It makes the model simple and efficient which in turn reduces the run time of the model. PCA prevents overfitting and converts a group of correlated variables to uncorrelated variables through orthogonal transformation^58^. A principal component (PC) is chosen such that it would describe most of the available variance^59^. Thus, it removes the risk of multicollinearity. In this study, the PCs of 21 GCMs of NEX-GDDP dataset and 13 GCMs of downscaled CMIP6 dataset for each grid was calculated separately. The PC’s which gave cumulative contribution rates greater than 95% were used as input to ML models.

### Machine learning algorithms

MMEs were developed for P, Tmax and Tmin separately at each grid point in the basin using machine learning methods. The observed and simulated values of P, Tmax and Tmin were divided into a calibration period and validation period. The first 45-years (1951–1995) of overlapping observed and simulated data were used for calibrating the MMEs. The rest of the data were used for validating the MMEs. More about the methods adopted in the study are given in the following sections.

#### Multiple linear regression (MLR)

MLR is a common form of regression analysis. Multiple linear regression attempts to explain the relationship between one dependent variable and two or more independent variables by fitting a linear Eq.^60^. It has been widely used for climate studies for downscaling and impact analysis^27,61^. In general, MLR can be mathematically written as:

1 $$y={\beta }_{0}+{\beta }_{1}{x}_{1}+\dots +{\beta }_{n}{x}_{n}+\varepsilon $$

where y is the dependent variable, ${\mathrm{x}}_{\mathrm{i}}$ are independent variables, ${\upbeta }_{\mathrm{i}}$ are parameters, $\upvarepsilon$ is the error.

In this study, the ordinary linear least squares (LLS) regression which minimizes the residual sum of squares between the observed values and the ensemble values was used. This was implemented using ‘sklearn.linear_model’ module in python^54^.

#### Support vector machine

SVM is based on Vapnik–Chervonenkis (VC) theory and the rule of structural risk minimization^62^. SVM is used for various climate change and hydrological applications^2,23,25,63^. Support Vector Regression (SVR) is the SVM that elucidates nonlinear regression problems by mapping the low-dimensional data to a high-dimensional feature space using kernel functions. Mathematically, SVR model can be represented as follows:

2 $$y=\sum_{i=1}^{n}\left({\alpha }_{i}-\widehat{{\alpha }_{i}}\right)Kernel\langle {x}_{i},x\rangle +b$$

where $\mathrm{Kernel}\langle {\mathrm{x}}_{\mathrm{i}},\mathrm{x}\rangle$ represents the kernel function used; ${\mathrm{\alpha }}_{\mathrm{i}}\,\,\mathrm{ and}\,\,\widehat{{\mathrm{\alpha }}_{\mathrm{i}}}$ denote the Lagrange multipliers; ${\mathrm{x}}_{\mathrm{i}}$ denote the vectors; x represents the independent vector; b represents the bias parameter.

SVR uses a symmetrical loss function, which equally penalizes high and low misestimates. Using Vapnik’s Open image in new window -insensitive approach, a flexible tube of minimal radius is formed symmetrically around the estimated function, such that the absolute values of errors less than a certain threshold Open image in new window are ignored both above and below the estimate. In this manner, points outside the tube are penalized, but those within the tube, either above or below the function, receive no penalty. One of the main advantages of SVR is that its computational complexity does not depend on the dimensionality of the input space. Additionally, it has excellent generalization capability, with high prediction accuracy^64^.

MMEs which used the polynomial kernel function performed better than the MMEs that used other kernel functions. Hence in this study polynomial kernel function was put to use similar to Sachindra et al.^23^ and Ahmed et al.^2^. The choice of hyperparameters plays a great role in machine learning methods. In the current study, the Bayesian hyperparameter optimization (BHO) was used to determine the hyperparameters for all machine learning algorithms. The “hyperopt” package in Python was used to implement BHO^65^. The important hyper-parameters optimized in SVR are C, kernel function and epsilon.

#### Random forest and extra tree regressor

The RF and ETR models are ensemble machine learning techniques. RF is proposed by Breiman^66^ based on a combination of statistical learning theory and classification or regression methods. The multiple classification and regression decision tree (CART) included in the algorithm prevents over-fitting and adjusts different types of input variables. This algorithm generates many independent trees and generates a decision based on the characteristics of nonparametric statistical regression and randomness^26^. A decision tree comprises of a root node, sub node, and leaf node. A leaf node corresponds to a judgement level while a sub node contains a judgement rule. The average of predicted values from all trees is the result of the algorithm. RF is internally cross-validated using out of bag (OOB) score^25^. ETR is a variation of that adds a further level of randomness to the splitting of the trees^67^. It is an extension of RF with two major differences: (1) ETR does not apply bootstrapping but each tree is trained with the whole of training data, (2) ETR selects a random cut point instead of a locally optimum cut point. The split which gives the highest score is selected from the set of randomly generated splits. That is k decision trees are generated and m features at selected for each training sample. At each of the decision tree a random cut-point is selected. This helps to avoid overfitting to some extent. More about ETR can be found in Xu et al.^25^.

#### Long short-term memory (LSTM) deep learning models

**Figure 2:**
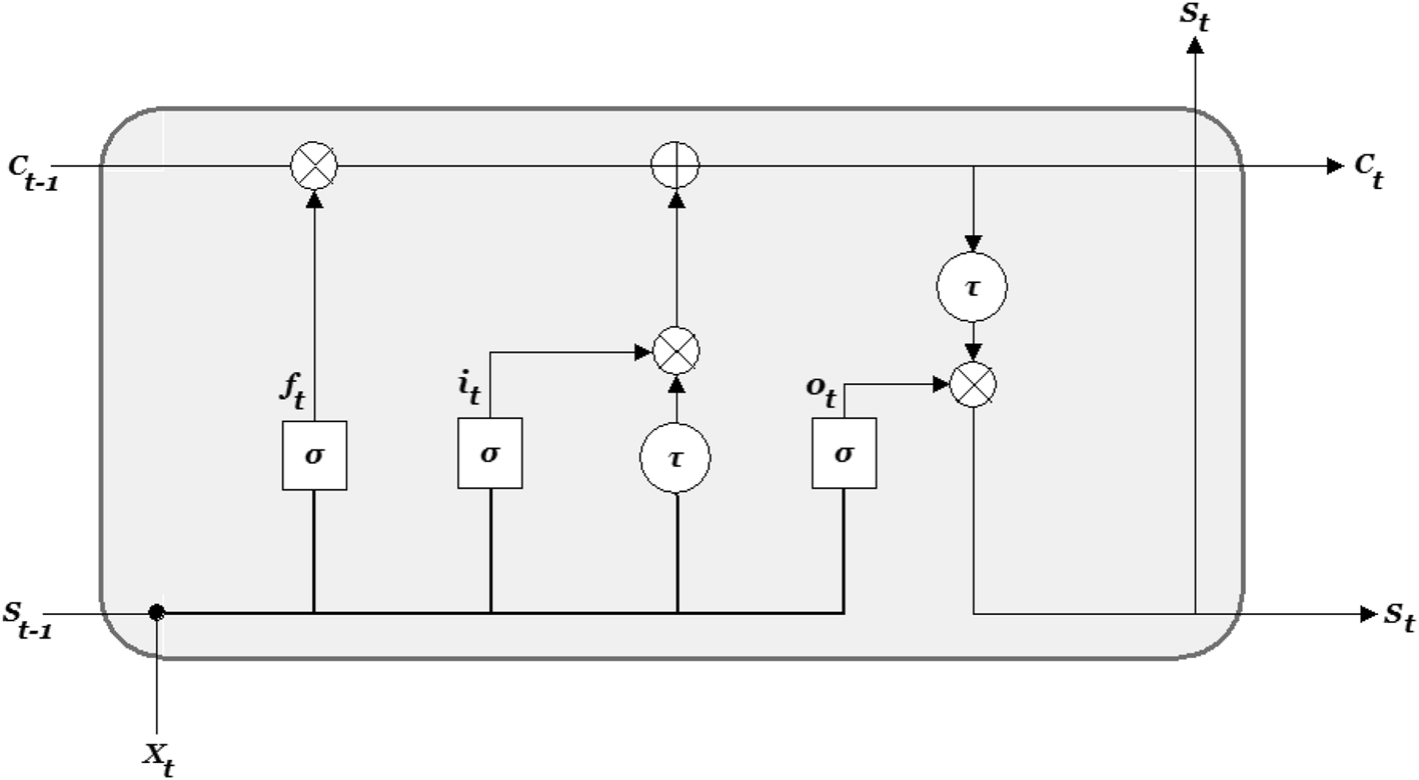
Architecture of a LSTM cell.

Climate data is a time series data involving sequence of observations over regularly spaced intervals with trend (upward, downward, or absent), seasonality (periodic fluctuation within a certain period), cyclic variations (rises and falls) and irregular or random components^68,69^. Meteorological predictions of GCMs can be seen as a multivariate sequential data. Hence a LSTM model which belongs to the family of deep recurrent neural networks could be used for creating multi-model ensembles of climate data. The current prediction of LSTMs is influenced by the feed network activations from the previous time steps. Hence, this connection develops a memory of previous events in the LSTM network. The architecture of a LSTM cell is given in Fig. 2 where f_t_, i_t_ and o_t_ are forget, input, and output gates respectively. X_t_, S_t_ and C_t_ are input, hidden and cell state at time step t, respectively. S_t-1_ and C_t-1_ are the hidden and cell state at time step t − 1, respectively.  ⊗ ,  ⊕  and σ are pointwise multiplication, pointwise addition and sigmoid activation, respectively.

The network has three inputs: X_t_—input at the current time step, S_t-1_ is the output from the previous LSTM unit, and C_t-1_ is the memory of the previous unit. As for outputs, S_t−_ the output of the current network, and C_t_ is the memory of the current unit. The LSTM model has input i_t_, output o_t_, and forget f_t_ learnable gates that modulate the flow of information and maintains an explicit hidden state that is recursively carried forward and updated as each element of the sequential data is passed through the network. The input gate decides what information to add from the present input to the cell state, the forget gate decides what must be removed from the S_t-1_ state, thus keeping only relevant information, and the output gate decides what information to output from the current cell state. More information LSTM can be found in Bouktif et al.^69^ and Sagheer and Kotb^70^. In this study, the LSTM was optimised for learning rate, batch size, units, layers and window.

### Performance evaluation

The observed and simulated values of P, Tmax and Tmin used for developing MMEs are divided into calibration and validation dataset. The first 45-years (1951–1995) of overlapping observed and simulated data were used for calibrating the machine learning models. The rest of the data were used for validating the MMEs developed using each method. Performance evaluation on validation data on daily basis was done in terms of Root-Mean Square Error (RMSE) or Root-Mean Square Deviation (RMSD) and correlation coefficient (R). These performance indicators are widely used by many researchers^71–73^. Further, the daily data were converted into monthly data for performance evaluation. Scatter plots and Taylor diagrams are used for the evaluation of performance on monthly basis. The scatter plots along with coefficient of determination (R^2^) provided a useful comparison of observed and MME values. Taylor diagram summarised the performance of each MME in terms of RMSD, R and standard deviation (SD). The procedure was repeated explicitly for MME’s of precipitation for the monsoon season to study their ability in simulating rainfall magnitudes.

## Results and discussion

The performance evaluation of each ensembling method for simulating P, Tmin and Tmax is done grid wise on daily and monthly scales for NEX-GDDP and CMIP6 datasets separately. The performance evaluation on daily scale is done using R and RMSE. Results of this evaluation during the validation period is given in Table 3. Further, scatter plots and Taylor diagrams are used to evaluate the performance on a monthly basis. The performance of each ML method was more or less the same at each grid. Hence, the results obtained for one representative grid in the basin is shown and discussed for simplification of the presentation.

**Table 3:** Performance of various MMEs in simulating daily P, Tmin and Tmax.

Methods	Precipitation		Minimum temperature		Maximum Temperature	
	R	RMSE	R	RMSE	R	RMSE
**NEX-GDDP dataset**						
Mean	0.519	19.03	0.522	1.78	0.484	2.33
MLR	0.552	18.55	0.828	1.15	0.838	1.45
SVM	0.565	18.37	0.835	1.15	0.832	1.47
ETR	0.567	18.33	0.836	1.15	0.860	1.35
RF	0.572	18.25	0.838	1.14	**0.872**	**1.30**
LSTM	**0.736**	**14.59**	**0.872**	**1.30**	0.868	1.32
**CMIP6 dataset**						
Mean	0.539	18.22	0.453	2.44	0.485	2.33
MLR	0.549	18.10	0.754	1.39	0.844	1.44
SVM	0.556	17.99	0.756	1.39	0.861	1.36
ETR	0.567	17.83	0.780	1.33	0.864	1.35
RF	0.577	17.68	0.781	1.33	0.864	1.35
LSTM	**0.728**	**14.56**	**0.801**	**1.27**	**0.869**	**1.33**

Significant values are in bold.

### Performance evaluation of MMEs in the case of precipitation

#### Performance evaluation of MMEs for daily rainfall

**Figure 3:**
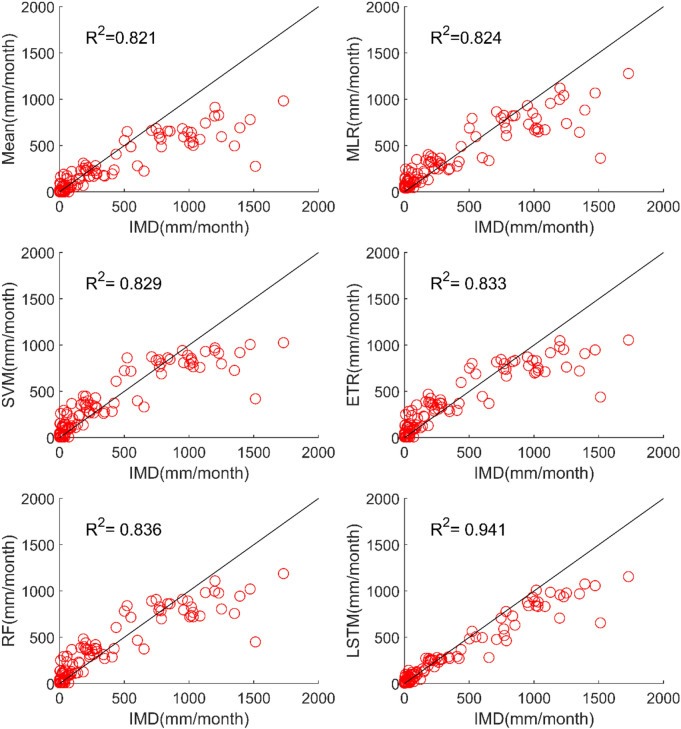
Scatter plot of observed and MME simulated monthly precipitation for NEX-GDDP dataset.

**Figure 4:**
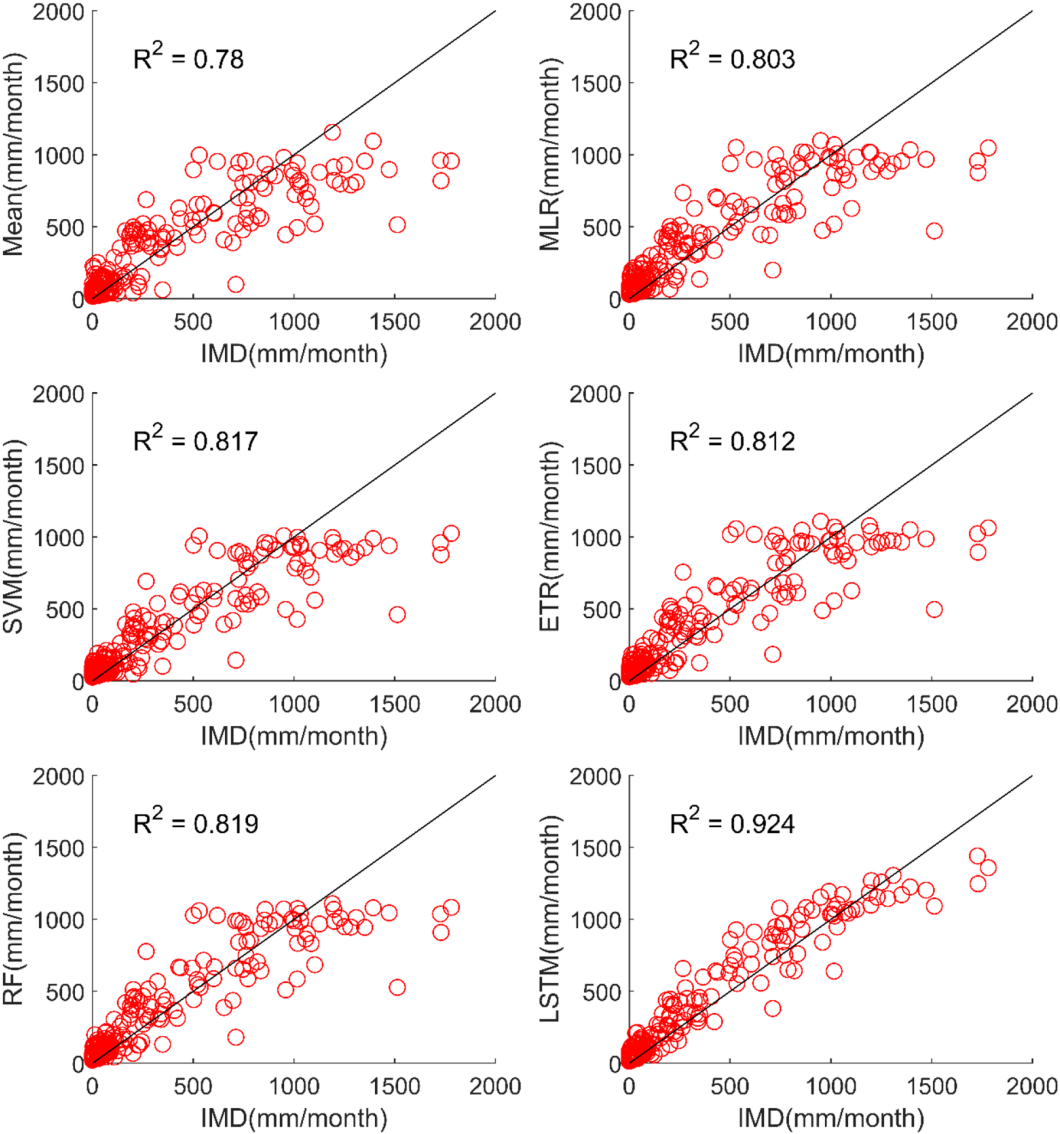
Scatter plot of observed and MME simulated monthly precipitation for CMIP6 dataset.

**Figure 5:**
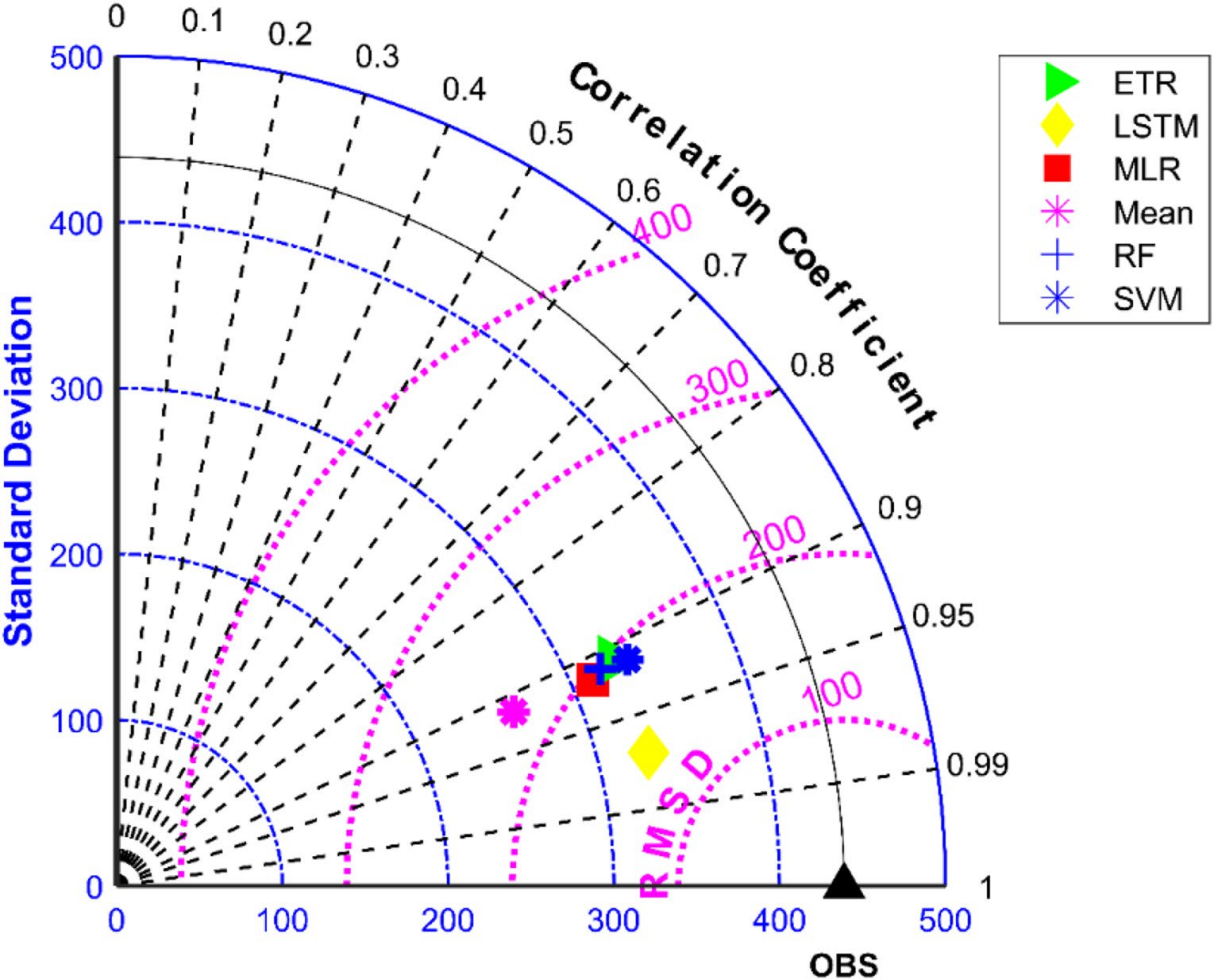
Taylor diagram of observed and MME simulated monthly precipitation of NEX-GDDP dataset during the validation period.

**Figure 6:**
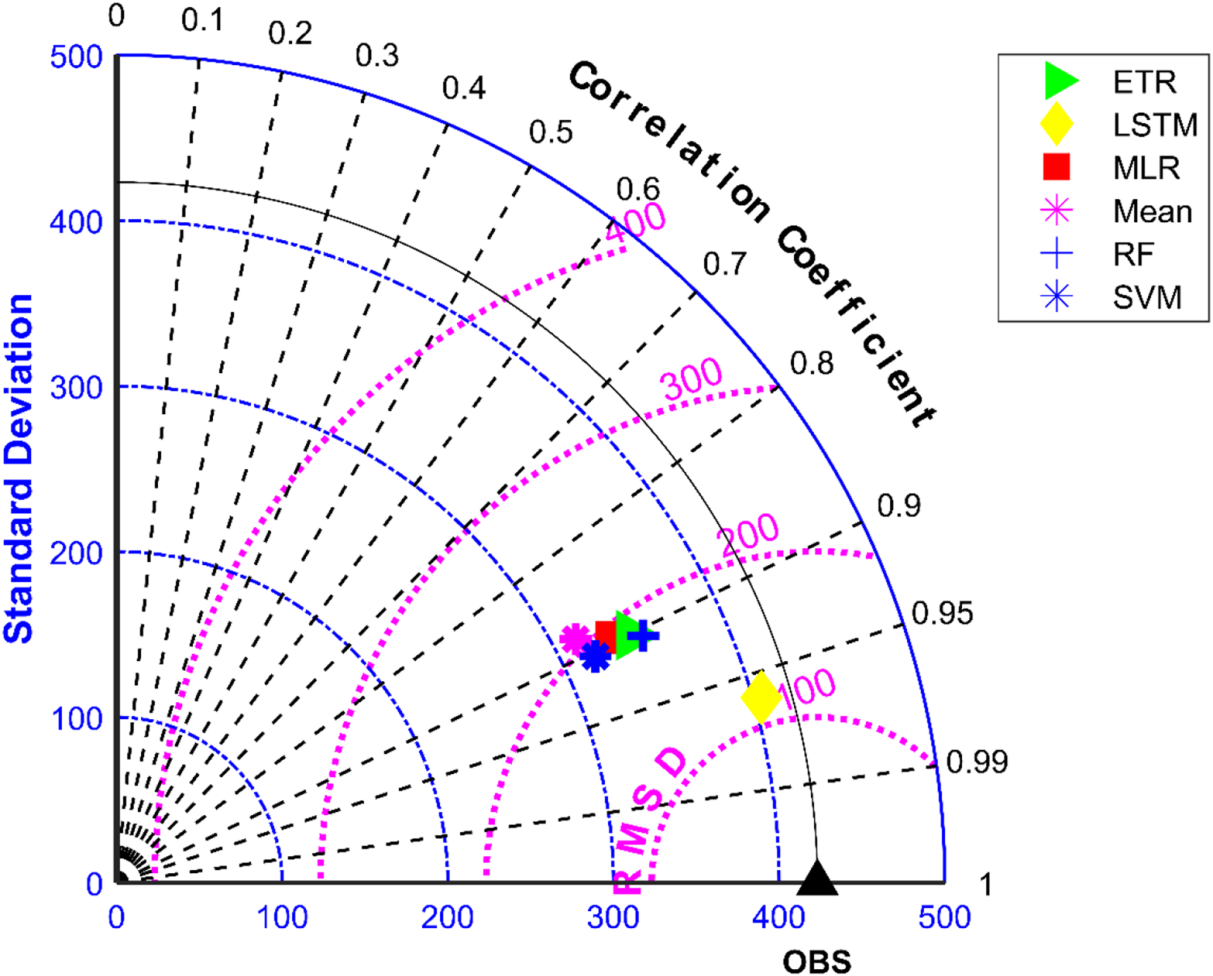
Taylor diagram of observed and MME simulated monthly precipitation of CMIP6 dataset during the validation period.

The results of performance evaluation on daily precipitation given in Table 3 indicate that the ML approaches have improved performance of MMEs when compared with the mean ensemble approach. However, the improvements are not very significant for all ML methods except for LSTM. The MME developed using LSTM for NEX-GDDP dataset could significantly improve the R value from 0.52 to 0.74 when compared to mean ensemble technique. Similarly, a reduction in RMSE from 19.03 to 14.59 is also achieved by using LSTM for ensembling when compared to mean ensembling. Thus, the MMEs made using LSTM is performing significantly better for NEX-GDDP and CMIP6 datasets. The same is observed in the scatterplots of monthly precipitation given in Figs. 3 and 4 for NEX-GDDP and CMIP6 datasets respectively. The R^2^ value increased from 0.82 to 0.94 and 0.78 to 0.92 for LSTM ensemble when compared to mean ensemble for NEX-GDDP and CMIP6 dataset respectively. Figures 5 and 6 show the Taylor diagrams of observed and MME simulated monthly precipitation of NEX-GDDP and CMIP6 datasets respectively for the validation period. These figures demonstrate that MME developed using LSTM method matches better with the observed data than MMEs developed using other methods.

#### Performance evaluation of MMEs for monsoon season

**Figure 7:**
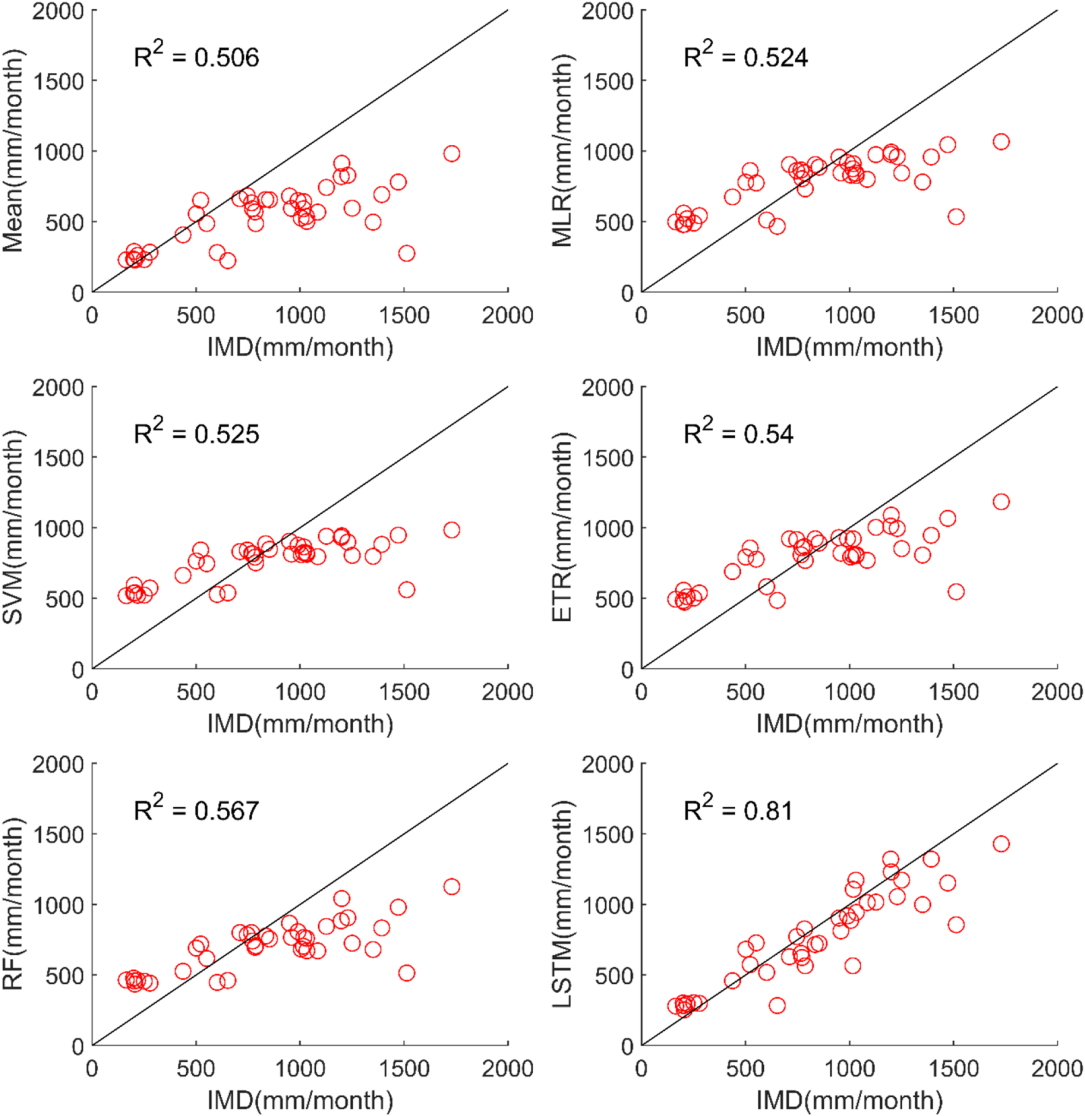
Scatter plot of observed and MME simulated monthly monsoon precipitation for NEX-GDDP dataset.

**Figure 8:**
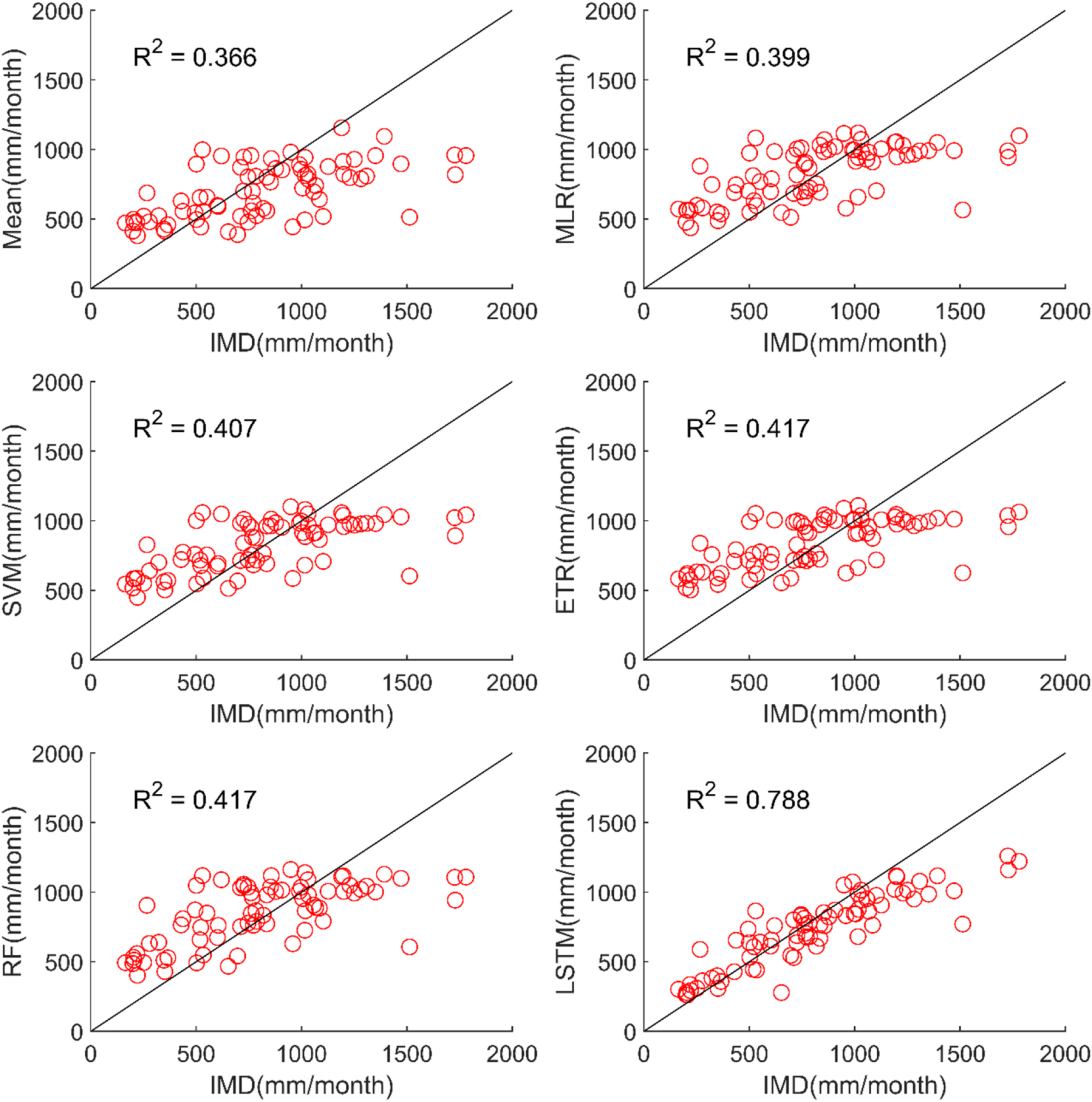
Scatter plot of observed and MME simulated monthly monsoon precipitation for CMIP6 dataset.

**Figure 9:**
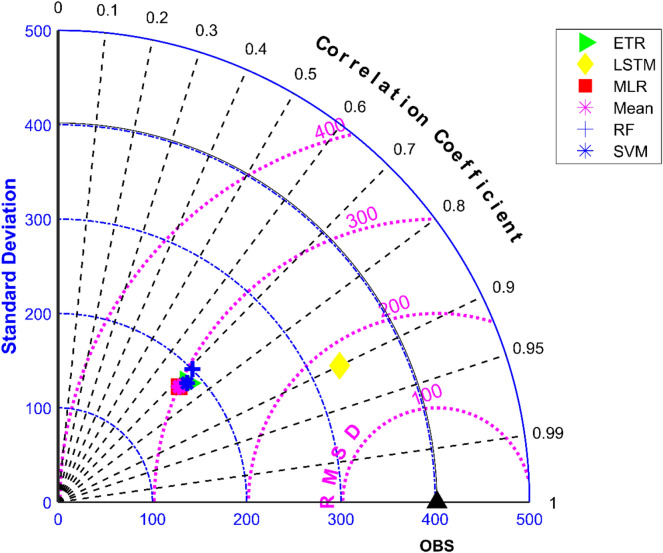
Taylor diagram of observed and MME simulated monthly monsoon precipitation of NEX-GDDP dataset during the validation period.

**Figure 10:**
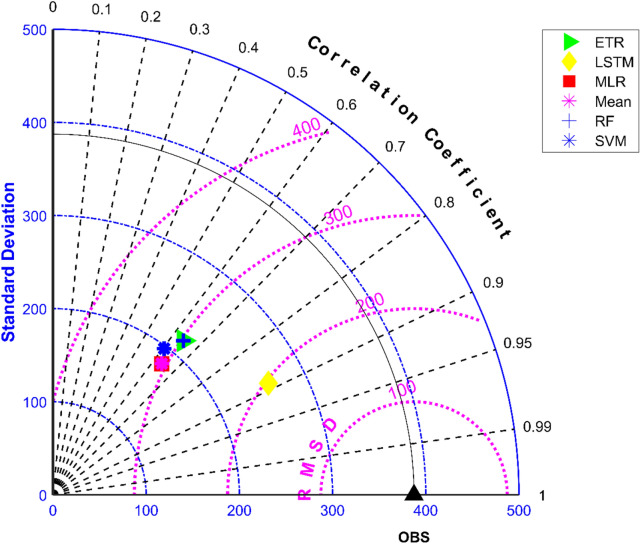
Taylor diagram of observed and MME simulated monthly monsoon precipitation of CMIP6 dataset during the validation period.

The results of performance evaluation on daily precipitation of monsoon months (June to September) are given in Table 4. These results indicate that the ML approaches namely, MLR, SVM, ETR and RF have shown very slight and insignificant improvement in performance of MMEs when compared with the mean ensemble approach in the case of daily precipitation in monsoon months of NEX-GDP and CMIP6 datasets. However, MME made using LSTM has shown significant improvement in the performance of daily monsoon rainfall in terms of R and RMSE. The MME developed using LSTM for NEX-GDDP dataset could improve the R value from 0.038 to 0.386 when compared to mean ensemble technique. Similarly, a reduction in RMSE from 31.49 to 23.35 is also achieved by using LSTM model. Similar improvements in R (0.031 to 0.357) and RMSE (29.26 to 23.33) was seen in the case of CMIP6 dataset. Thus, the MMEs of monsoon precipitaion made using LSTM is performing significantly better for NEX-GDDP and CMIP6 datasets. The same is observed in the scatterplots of monthly precipitation given in Figs. 7 and 8 for NEX-GDDP and CMIP6 datasets respectively. The R^2^ value increased from 0.506 to 0.81 and 0.366 to 0.788 for LSTM ensemble when compared to mean ensemble for NEX-GDDP and CMIP6 dataset respectively. Figures 9 and 10 show the Taylor diagrams of observed and MME simulated monthly monsoon precipitation of NEX-GDDP and CMIP6 datasets respectively for the validation period. These figures demonstrate that MME of monsoon precipitation developed using LSTM method matches better with the observed data than MMEs developed using other methods.

**Table 4:** Performance of various MMEs in simulating monsoon P.

Methods	Precipitation	
	R	RMSE
**NEX-GDDP dataset**		
Mean	0.038	31.49
MLR	0.042	30.25
SVM	0.053	30.08
ETR	0.065	29.89
RF	0.069	29.82
LSTM	0.386	23.35
**CMIP6 dataset**		
Mean	0.031	29.26
MLR	0.043	29.08
SVM	0.053	28.93
ETR	0.061	28.81
RF	0.061	28.82
LSTM	0.357	23.33

### Performance evaluation of MMEs in the case of maximum temperature

**Figure 11:**
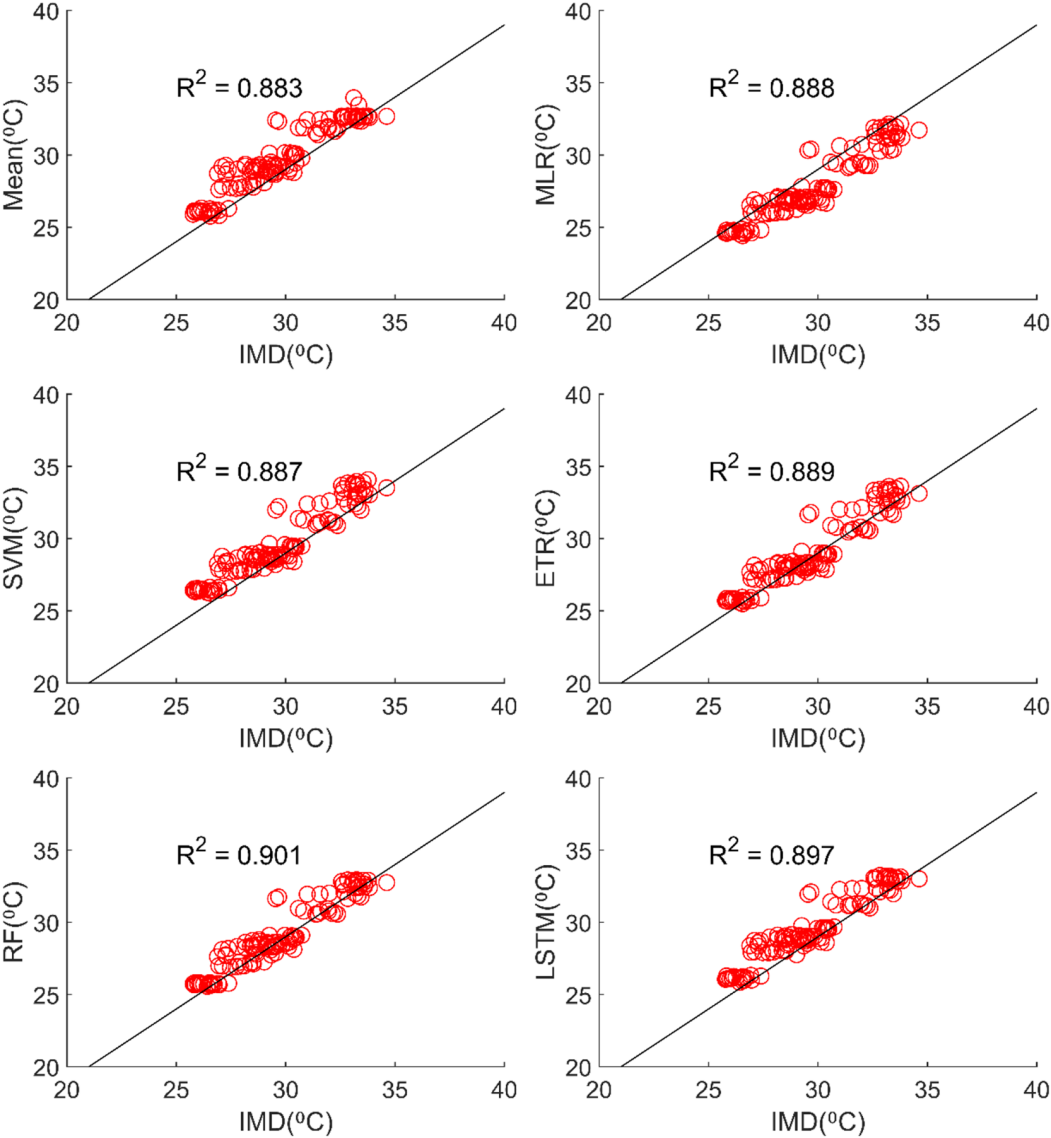
Scatter plot of observed and MME simulated average monthly maximum temperature for NEX-GDDP dataset.

**Figure 12:**
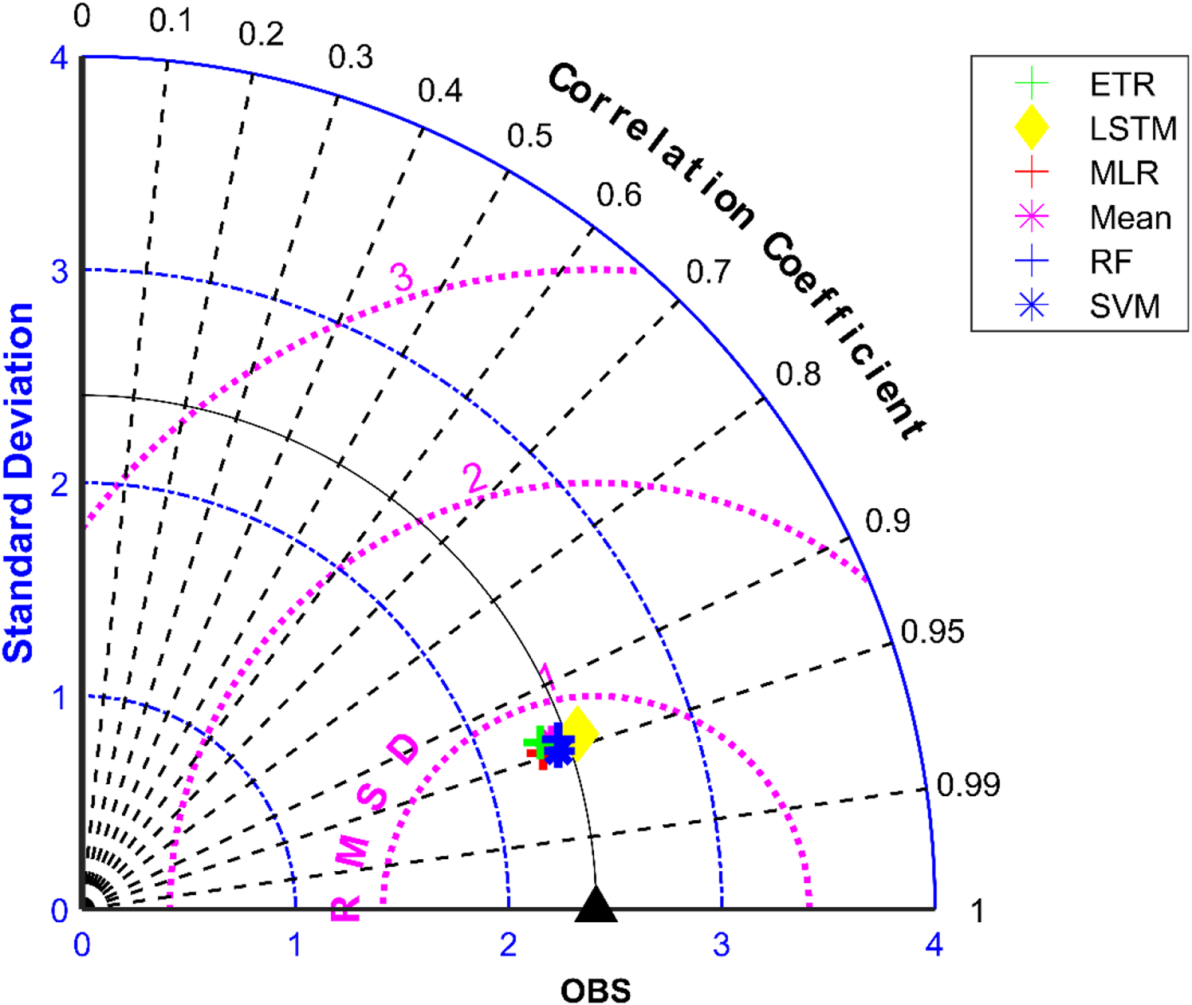
Taylor diagram of observed and MME simulated average monthly maximum temperature of NEX-GDDP dataset during the validation period.

**Figure 13:**
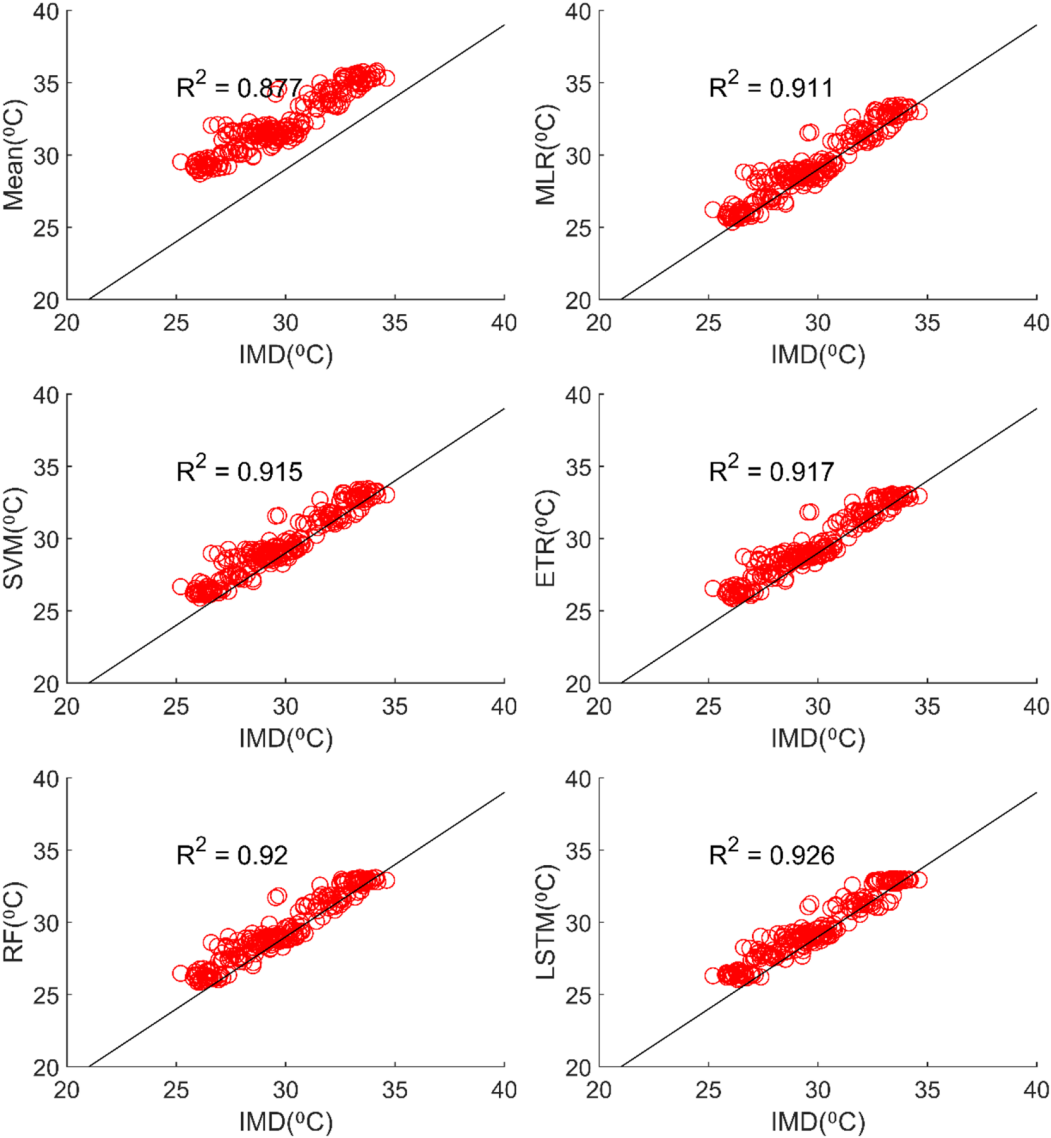
Scatter plot of observed and MME simulated average monthly maximum temperature for CMIP6 dataset.

**Figure 14:**
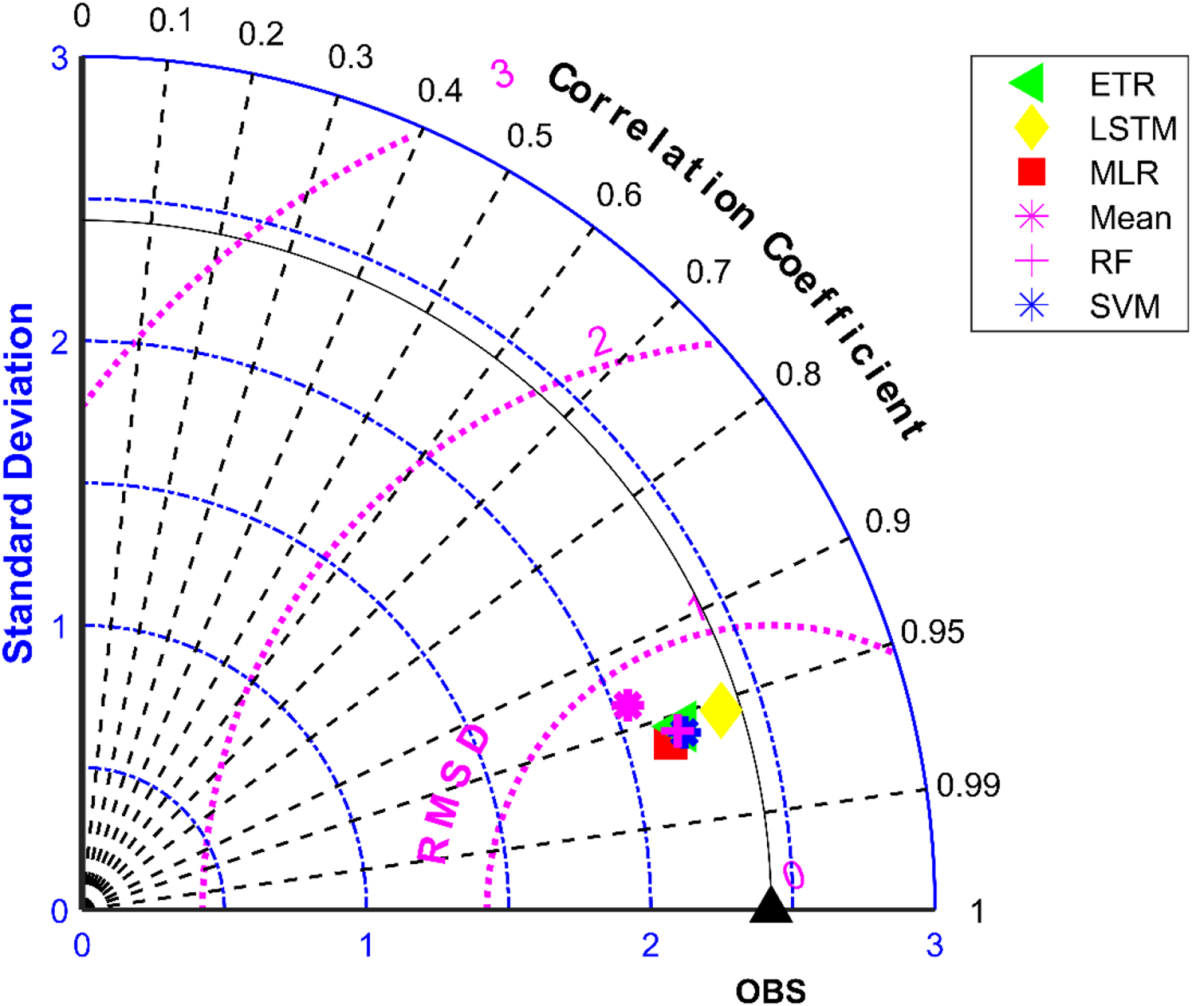
Taylor diagram of observed and MME simulated average monthly maximum temperature of CMIP6 dataset during the validation period.

Table 3 reveals that all ML methods performed significantly better in simulating daily maximum temperature when compared to ensemble mean approach. The MMEs developed for NEX-GDDP dataset using MLR, SVM, ETR, RF and LSTM gave R values of 0.838, 0.832, 0.86, 0.872 and 0.868 respectively while mean ensemble gave a R value of 0.484. The MMEs made using LSTM and RF method performed the best with RF slightly outperforming LSTM. Further, the MLR method slightly outperformed SVM method. The Figs. 11 and 12 shows the scatter plot and Taylor diagram of average monthly maximum temperature simulations of MMEs developed by different ensembling approaches against reference dataset. These figures show the performance of MMEs developed by all ensembling methods are more or less the same on a monthly basis. In the case of CMIP6 dataset significant improvement is seen in the MMEs developed by ML methods when compared to mean ensemble approach on a daily and monthly case. MME developed by LSTM method performed the best with R value of 0.869 in the case of daily maximum temperature. The scatterplot (Fig. 13) and Taylor diagram (Fig. 14) show the better performance of all ML methods when compared to mean ensemble approach.

### Performance evaluation of MMEs in the case of minimum temperature

**Figure 15:**
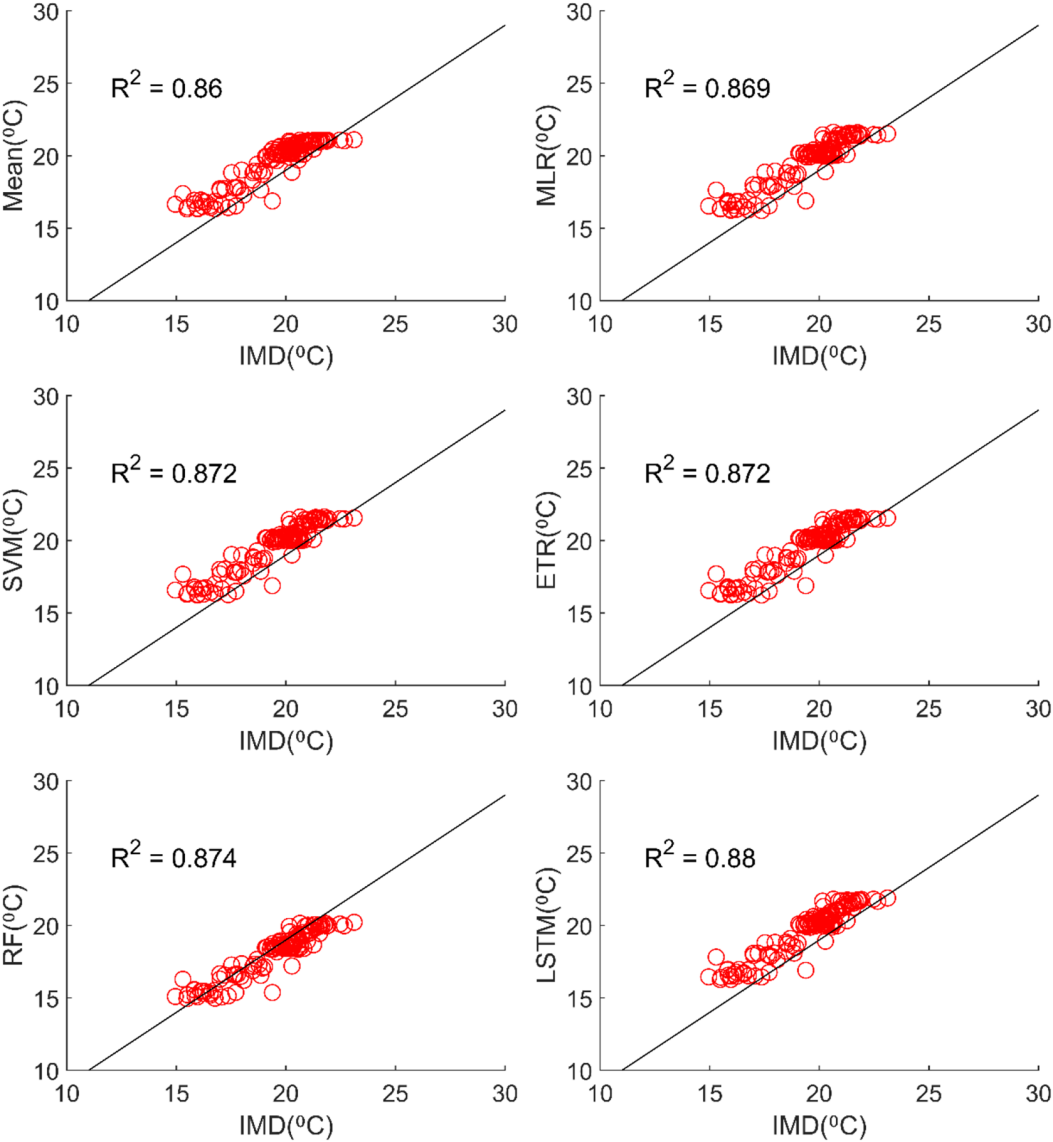
Scatter plot observed and MME simulated average monthly minimum temperature for NEX-GDDP dataset.

**Figure 16:**
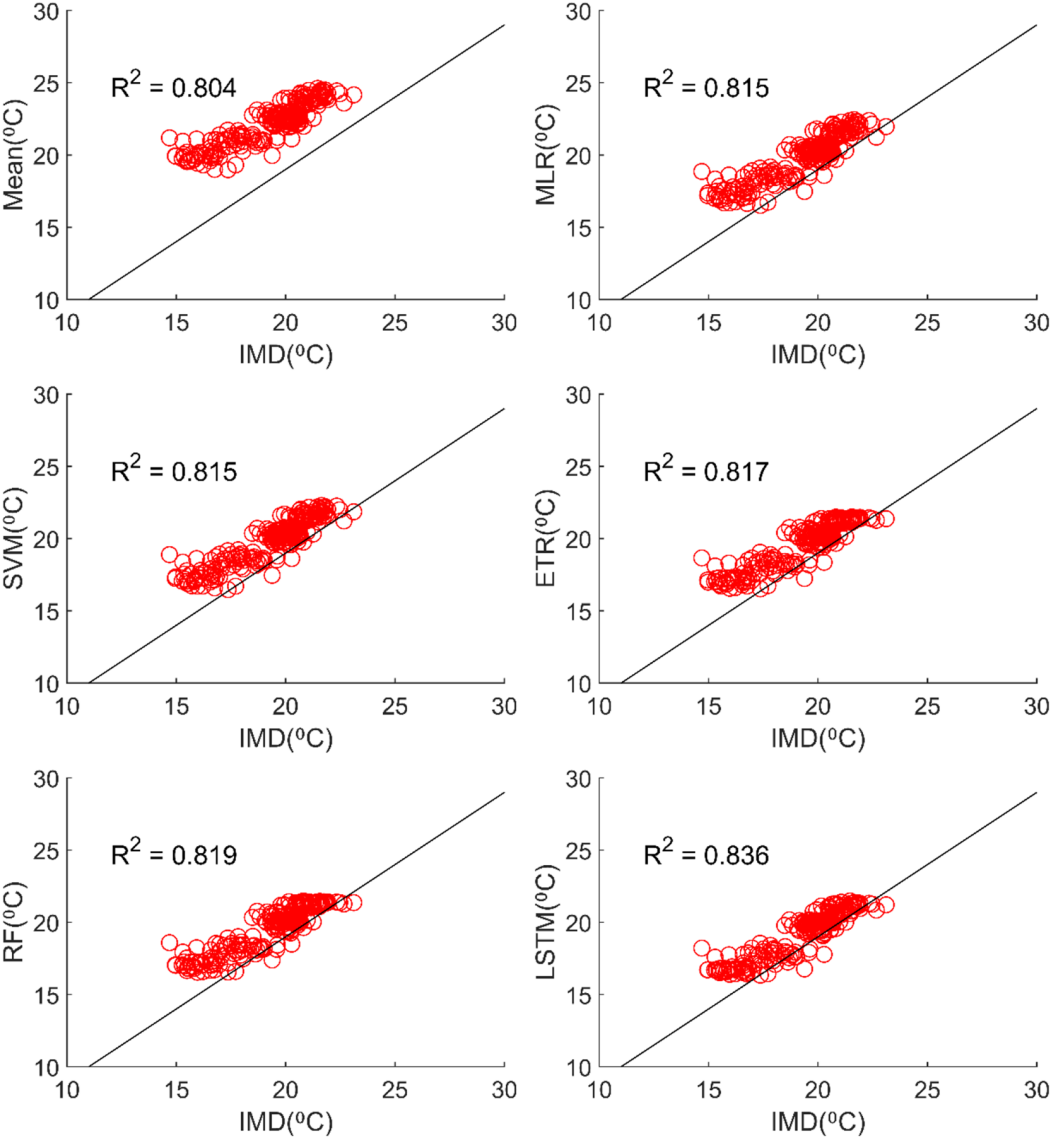
Scatter plot of observed and MME simulated average monthly minimum temperature for CMIP6 dataset.

**Figure 17:**
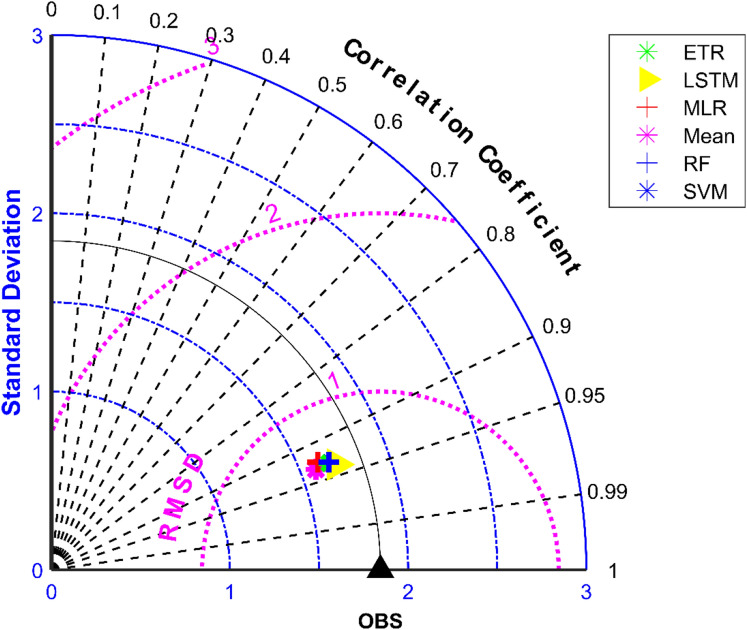
Taylor diagram of observed and MME simulated average monthly minimum temperature of NEX-GDDP dataset during the validation period.

**Figure 18:**
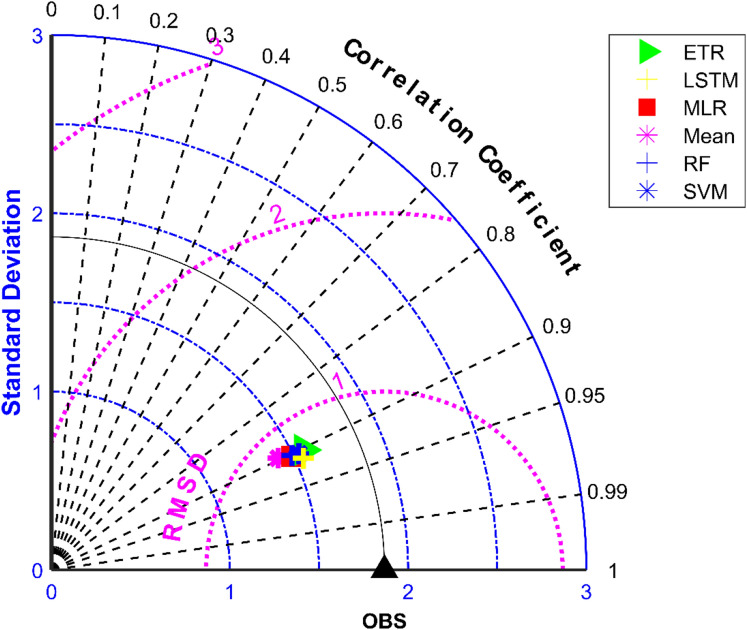
Taylor diagram of observed and MME simulated average monthly minimum temperature of CMIP6 dataset during the validation period.

All ML methods performed significantly better than mean ensembling methods in the case of minimum temperature in the case of NEX-GDDP and CMIP6 datasets. In the case of NEX-GDDP dataset the R value improved from 0.522 to 0.8 when ML methods are used. A similar increase in R value was also observed for CMIP6 dataset. When it came to evaluation of average monthly minimum temperature no significant improvement is observed. This can be observed in the scatter plots and Taylor diagrams. Figures 15 and 16 show the scatter plots of different MMEs of average monthly minimum temperature against reference dataset for NEX-GDDP and CMIP6 dataset respectively. Figures 17 and 18 show the Taylor diagrams of various MMEs developed for average monthly minimum temperature. However, LSTM remained to be the best performing model in the case of minimum temperature with R values of 0.872 and 0.801 for NEX-GDDP and CMIP6 datasets.

### Inter-comparisons of performance of different MMEs

Different approaches like mean, regression models (i.e., SVM and MLR), an ensemble learning models (i.e., ETR and RF), and deep learning time series model (i.e., multivariate LSTM) are used to create MMEs for 21 NEX-GDDP models and 13 CMIP6 models outputs for P, Tmin and Tmax. In the case of precipitation LSTM significantly outperformed all the other MME approaches with R values of 0.74 and 0.73 for NEX-GDDP and CMIP6 dataset respectively. The performance of all the other MME approaches was more or less the same with R values in the range of 0.52 to 0.58. Similarly, MMEs of LSTM gave R^2^ of 0.94 and 0.92 in the case of NEX-GDDP and CMIP6 datasets for monthly precipitation. The study done explicitly for monsoon rainfall shows that all methods except LSTM failed in giving good performance of MMEs. This shows that LSTM method to an extend is successful in predicting rainfall magnitude in monsoon months. Hence, this study reveals the superiority of LSTM compared to other methods in ensembling monsoon precipitation.

However, in the case of temperature, all ML approaches performed equally good when compared to mean ensembling approach. All ML methods could improve the R value from 0.5 to a range of 0.8 in the case of temperature. In the case of maximum temperature of NEX-GDDP dataset, the MME made with RF (R = 0.872) slightly outperformed LSTM (R = 0.868). In all the other cases of all ML methods performed equally well, with LSTM showing a slightly increased performance. The same pattern was observed in all the grid points in the basin. Ensemble learning models like RF and ETR also performed well in the basin in the case of maximum and minimum temperature. They outperformed MLR and SVM in all the cases. Hence, MMEs developed through LSTM, RF and ETR algorithms are recommended for creating MMEs in the basin. In general, all ML methods performed better than mean ensemble approach. This is seen in other studies like that of Ahmed et al.^2^.

## Summary and conclusions

In this study, an attempt has been taken to evaluate the performance of MMEs developed using six ensembling methods. These ensembling techniques include simple statistical technique (mean), regression models (i.e., SVM and MLR), ensemble learning models (i.e., ETR and RF), and deep learning time series model (i.e., LSTM). The performance evaluation of each ensembling technique was done in order to find the best-performing MMEs of 21 NEX-GDDP and 13 CMIP6 GCMs in Netravati basin. This comparison shows that the application of a LSTM model for climate model ensemble prediction performs significantly better than the benchmark models including other machine learning techniques and mean ensembling techniques in the case of precipitation. It gave a coefficient of determination of 0.94 and 0.92 in the case of NEX-GDDP and CMIP6 monthly precipitation datasets. The MME of LSTM method could simulate the monsoon rainfall magnitude satisfactorily when compared to all the other methods. Hence, LSTM deep learning models are seen to be an attractive approach for climate data prediction. This could be because of its capability in learning long-term dependencies in observed data, which lead to better predictions results that outperform several alternative machine learning and statistical approaches. In case of temperature all the ML methods showed equally good performance, with RF and LSTM performing consistently well in all the cases of temperature. The coefficient of determination in the range of 0.9 and 0.8 are observed for MMEs developed using RF and LSTM techniques in the case of monthly average maximum and minimum temperature respectively. Hence, based on this study RF and LSTM are recommended for creation of MMEs in the basin. In general, all ML approaches performed better than mean ensemble approach. However, this study limits its scope to machine learning methods and does not analyse its performance on extreme vales. Hence, a future study which analyses its effectiveness on extreme values may be done. Further, other multi-model combination like triple collocation and Bayesian approaches may be explored in future studies^53,74,74^. Thus, based on present study the following specific conclusions may be drawn:

1. The inter-comparison of MMEs developed using mean, SVM, MLR, ETR, RF and LSTM show that ML-based MMEs performed better than the mean ensemble approach. Therefore, ML methods are recommended for the creation of MMEs of climate data in future studies.
2. A time series model like LSTM could be a good choice for creation of MMEs. Hence, more studies which explore the usage of time series/sequential models for creation of MMEs may be done in the future.

## Data Availability

The daily gridded precipitation, maximum temperature and minimum temperature data can be accessed through IMD Pune’s website (http://www.imdpune.gov.in/Clim_Pred_LRF_New/Grided_Data_Download.html). The NEX-GDDP dataset used can be accessed from NASA Centre for Climate Simulation (NCCS) portal (https://portal.nccs.nasa.gov/datashare/NEXGDDP/). The downscaled CMIP6 data used in this study is made available by Mishra et al.^51^ at http://doi.org/10.5281/zenodo.3874046.

## References

[1] Nilawar AP, Waikar ML (2019). Impacts of climate change on streamflow and sediment concentration under RCP 4.5 and 8.5: a case study in Purna river basin, India. Sci. Total Environ..

[2] Ahmed K (2020). Multi-model ensemble predictions of precipitation and temperature using machine learning algorithms. Atmos. Res..

[3] Raju KS, Kumar DN (2020). Review of approaches for selection and ensembling of GCMs. J. Water Clim. Chang.

[4] Taylor KE, Stouffer RJ, Meehl GA (2012). An overview of CMIP5 and the experiment design. Bull. Am. Meteorol. Soc..

[5] Jose DM, Dwarakish GS (2020). Uncertainties in predicting impacts of climate change on hydrology in basin scale : a review. Arab. J. Geosci..

[6] Brown C (2014). Analysing uncertainties in climate change impact assessment across sectors and scenarios. Clim. Change.

[7] Chokkavarapu N, Mandla VR (2019). Comparative study of GCMs, RCMs, downscaling and hydrological models: a review toward future climate change impact estimation. SN Appl. Sci..

[8] Jose DM, Dwarakish GS (2022). Bias Correction and trend analysis of temperature data by a high-resolution CMIP6 Model over a Tropical River Basin. Asia-Pacific J. Atmos. Sci..

[9] Venkatesh K, Srinivas K, Preethi K (2020). Evaluation and integration of reanalysis rainfall products under contrasting climatic conditions in India. Atmos. Res..

[10] Pathak AA, Dodamani BM (2019). Comparison of meteorological drought indices for different climatic regions of an Indian River Basin. Asia-Pacific J. Atmos. Sci..

[11] Fowler HJ, Blenkinsop S, Tebaldi C (2007). Linking climate change modelling to impacts studies : recent advances in downscaling techniques for hydrological modelling. Int. J. Climatol..

[12] Laflamme EM, Linder E, Pan Y (2016). Statistical downscaling of regional climate model output to achieve projections of precipitation extremes. Weather Clim. Extrem. J..

[13] Piani C (2010). Statistical bias correction of global simulated daily precipitation and temperature for the application of hydrological models. J. Hydrol..

[14] Mudbhatkal A, Mahesha A (2018). Bias correction methods for hydrologic impact studies over India’s Western Ghat Basins. J. Hydrol. Eng..

[15] Singh A, Sahoo RK, Nair A, Mohanty UC, Rai RK (2017). Assessing the performance of bias correction approaches for correcting monthly precipitation over India through coupled models. Meteorol. Appl..

[16] Jose DM, Dwarakish GS (2021). Bias correction and trend analysis of temperature data by a high-resolution CMIP6 model over a tropical river Basin. Asia-Pacific J. Atmos. Sci..

[17] Jose DM, Dwarakish GS (2022). Ranking of downscaled CMIP5 and CMIP6 GCMs at a basin scale: case study of a tropical river basin on the South West coast of India. Arab. J. Geosci..

[18] Kundzewicz ZW (2018). Uncertainty in climate change impacts on water resources. Environ. Sci. Policy.

[19] Pavan V, Doblas-Reyes FJ (2000). Multi-model seasonal hindcasts over the Euro-Atlantic: Skill scores and dynamic features. Clim. Dyn..

[20] Gleckler PJ, Taylor KE, Doutriaux C (2008). Performance metrics for climate models. J. Geophys. Res. Atmos..

[21] Acharya N, Shrivastava NA, Panigrahi BK, Mohanty UC (2014). Development of an artificial neural network based multi-model ensemble to estimate the northeast monsoon rainfall over south peninsular India: an application of extreme learning machine. Clim. Dyn..

[22] Crawford J, Venkataraman K, Booth J (2019). Developing climate model ensembles: a comparative case study. J. Hydrol..

[23] Sachindra DA, Ahmed K, Rashid MM, Shahid S, Perera BJC (2018). Statistical downscaling of precipitation using machine learning techniques. Atmos. Res..

[24] Wang X, Yang T, Li X, Shi P, Zhou X (2017). Spatio-temporal changes of precipitation and temperature over the Pearl River basin based on CMIP5 multi-model ensemble. Stoch. Environ. Res. Risk Assess..

[25] Xu R, Chen N, Chen Y, Chen Z (2020). Downscaling and projection of multi-cmip5 precipitation using machine learning methods in the upper han river Basin. Adv. Meteorol..

[26] Xu L (2019). Improving the North American multi-model ensemble (NMME) precipitation forecasts at local areas using wavelet and machine learning. Clim. Dyn..

[27] Pang B, Yue J, Zhao G, Xu Z (2017). Statistical downscaling of temperature with the random forest model. Adv. Meteorol..

[28] Xu R, Chen Y, Chen Z (2019). Future changes of precipitation over the Han River basin using NEX-GDDP dataset and the SVR_QM method. Atmosphere (Basel)..

[29] Anderson GJ, Lucas DD (2018). machine learning predictions of a multiresolution climate model ensemble. Geophys. Res. Lett..

[30] Nourani V, Uzelaltinbulat S, Sadikoglu F, Behfar N (2019). Artificial intelligence based ensemble modeling for multi-station prediction of precipitation. Atmosphere (Basel)..

[31] Wang B (2018). Using multi-model ensembles of CMIP5 global climate models to reproduce observed monthly rainfall and temperature with machine learning methods in Australia. Int. J. Climatol..

[32] Kolluru V, Kolluru S, Wagle N, Acharya TD (2020). Secondary Precipitation estimate merging using machine learning: development and evaluation over Krishna River Basin, India. Remote Sens..

[33] Khashei M, Bijari M (2010). An artificial neural network (p, d, q) model for timeseries forecasting. Expert Syst. Appl..

[34] Hochreiter S, Schmidhuber J (1997). Long short-term memory. Neural Comput..

[35] Najafabadi MM (2015). Deep learning applications and challenges in big data analytics. J. Big Data.

[36] Alom MZ (2019). A state-of-the-art survey on deep learning theory and architectures. Electron..

[37] Myers N, Mittermeier RA, Mittermeier CG, Fonseca GAB, Kent J (2000). Biodiversity hotspots for conservation priorities. Nature.

[38] Mudbhatkal A, Mahesha A (2017). Regional climate trends and topographic influence over the Western Ghat catchments of India. Int. J. Climatol..

[39] Sinha RK, Eldho TI (2018). Effects of historical and projected land use/cover change on runoff and sediment yield in the Netravati river basin, Western Ghats, India. Environ. Earth Sci..

[40] Pai DS (2014). Development of a new high spatial resolution (025° × 025°) long period (1901–2010) daily gridded rainfall data set over India and its comparison with existing data sets over the region. Mausam.

[41] Srivastava AK, Rajeevan M, Kshirsagar SR (2009). Development of a high resolution daily gridded temperature data set (1969–2005) for the Indian region. Atmos. Sci. Lett..

[42] Bao Y, Wen X (2017). Projection of China’s near- and long-term climate in a new high-resolution daily downscaled dataset NEX-GDDP. J. Meteorol. Res..

[43] Raghavan SV, Hur J, Liong SY (2018). Evaluations of NASA NEX-GDDP data over Southeast Asia: present and future climates. Clim. Change.

[44] Singh V, Sharma A, Goyal MK (2019). Projection of hydro-climatological changes over eastern Himalayan catchment by the evaluation of RegCM4 RCM and CMIP5 GCM models. Hydrol. Res..

[45] Yu R, Zhai P, Lu Y (2018). Implications of differential effects between 1.5 and 2 °C global warming on temperature and precipitation extremes in China’s urban agglomerations. Int. J. Climatol..

[46] Wood AW, Leung LR, Sridhar V, Lettenmaier DP (2004). Hydrologic implications of dynamical and statistical approaches to downscaling climate model outputs. Clim. Change.

[47] Jain S, Salunke P, Mishra SK, Sahany S, Choudhary N (2019). Advantage of NEX-GDDP over CMIP5 and CORDEX Data: Indian Summer Monsoon. Atmos. Res..

[48] Singh V, Xiaosheng Q (2019). Data assimilation for constructing long-term gridded daily rainfall time series over Southeast Asia. Clim. Dyn..

[49] Zaman M, Fang G, Mehmood K, Saifullah M (2015). Trend change study of climate variables in Xin’anjiang-Fuchunjiang watershed. China. Adv. Meteorol..

[50] Singh V, Jain SK, Singh PK (2019). Inter-comparisons and applicability of CMIP5 GCMs, RCMs and statistically downscaled NEX-GDDP based precipitation in India. Sci. Total Environ..

[51] Mishra V, Bhatia U, Tiwari AD (2020). Bias-corrected climate projections for South Asia from Coupled Model Intercomparison Project-6. Sci. Data.

[52] Xu L, Chen N, Zhang X, Chen Z (2018). An evaluation of statistical, NMME and hybrid models for drought prediction in China. J. Hydrol..

[53] Xu L, Chen N, Zhang X, Chen Z (2020). A data-driven multi-model ensemble for deterministic and probabilistic precipitation forecasting at seasonal scale. Clim. Dyn..

[54] Pedregosa F (2011). Scikit-learn: machine learning in Python. J. Mach. Learn. Res..

[55] Chollet, F. *Deep learning with Python*. vol. 361 (Manning New York, 2018).

[56] Jollife IT, Cadima J (2016). Principal component analysis: a review and recent developments. Philos. Trans. R. Soc. A.

[57] Hotelling H (1933). Analysis of a complex of statistical variables into Principal Components. J. Educ. Psychol..

[58] Ayar PV (2016). Intercomparison of statistical and dynamical downscaling models under the EURO- and MED-CORDEX initiative framework: present climate evaluations. Clim. Dyn..

[59] Benestad R, Parding K, Dobler A, Mezghani A (2017). A strategy to effectively make use of large volumes of climate data for climate change adaptation. Clim. Serv..

[60] Uyanık GK, Güler N (2013). A study on multiple linear regression analysis. Procedia Soc. Behav. Sci..

[61] Themeßl MJ, Gobiet A, Leuprecht A (2011). Empirical-statistical downscaling and error correction of daily precipitation from regional climate models. Int. J. Climatol..

[62] Vapnik, V. *The Nature of Statistical Learning*. *Springer Science & Business Media* (Springer science & business media, 1995).

[63] Raghavendra S, Deka PC (2014). Support vector machine applications in the field of hydrology: a review. Appl. Soft Comput. J..

[64] Awad, M. & Khanna, R. Support Vector Regression BT - Efficient Learning Machines: Theories, Concepts, and Applications for Engineers and System Designers. in (eds. Awad, M. & Khanna, R.) 67–80 (Apress, 2015). 10.1007/978-1-4302-5990-9_4.

[65] Bergstra J, Komer B, Eliasmith C, Yamins D, Cox DD (2015). Hyperopt: A Python library for model selection and hyperparameter optimization. Comput. Sci. Discov..

[66] Breiman L (2001). Random forests. Mach. Learn..

[67] Geurts P, Ernst D, Wehenkel L (2006). Extremely randomized trees. Mach. Learn..

[68] Mudelsee M (2019). Trend analysis of climate time series: a review of methods. Earth-Sci. Rev..

[69] Bouktif S, Fiaz A, Ouni A, Serhani MA (2020). Multi-sequence LSTM-RNN deep learning and metaheuristics for electric load forecasting. Energies.

[70] Sagheer A, Kotb M (2019). Unsupervised pre-training of a deep LSTM-based stacked autoencoder for multivariate time series forecasting problems. Sci. Rep..

[71] Bhatti HA, Rientjes T, Haile AT, Habib E, Verhoef W (2016). Evaluation of bias correction method for satellite-based rainfall data. Sensors (Switzerland).

[72] Mendez M, Maathuis B, Hein-Griggs D, Alvarado-Gamboa LF (2020). Performance evaluation of bias correction methods for climate change monthly precipitation projections over Costa Rica. Water (Switzerland).

[73] Nyunt CT, Koike T, Yamamoto A (2016). Hydrol. Earth Syst. Sci. Discuss..

[74] Xu L (2021). In-situ and triple-collocation based evaluations of eight global root zone soil moisture products. Remote Sens. Environ..

[75] Mishra V, Bhatia U, Tiwari AD (2020). Zenodo.

